# Nursing Genetic Research: New Insights Linking Breast Cancer Genetics and Bone Density

**DOI:** 10.3390/healthcare8020172

**Published:** 2020-06-15

**Authors:** Antonio Sanchez-Fernandez, Raúl Roncero-Martin, Jose M. Moran, Jesus Lavado-García, Luis Manuel Puerto-Parejo, Fidel Lopez-Espuela, Ignacio Aliaga, María Pedrera-Canal

**Affiliations:** 1 Servicio de Tocoginecología, Complejo Hospitalario de Cáceres, 10004 Cáceres, Spain; gineantonio@gmail.com; 2 Metabolic Bone Diseases Research Group, Nursing Department, Nursing and Occupational Therapy College, University of Extremadura, Avd. Universidad s/n, 10003 Cáceres, Spain; rronmar@unex.es (R.R.-M.); jmlavado@unex.es (J.L.-G.); lmpuerto@unex.es (L.M.P.-P.); fidellopez@unex.es (F.L.-E.); mariapedreracanal@gmail.com (M.P.-C.); 3 Departamento de Estomatología II, Universidad Complutense de Madrid, 28040 Madrid, Spain; i.aliaga@pdi.ucm.es

**Keywords:** nursing, genetics, bone density, GREB1

## Abstract

Nursing research is expected to provide options for the primary prevention of disease and health promotion, regardless of pathology or disease. Nurses have the skills to develop and lead research that addresses the relationship between genetic factors and health. Increasing genetic knowledge and research capacity through interdisciplinary cooperation as well as the development of research resources, will accelerate the rate at which nurses contribute to the knowledge about genetics and health. There are currently different fields in which knowledge can be expanded by research developed from the nursing field. Here, we present an emerging field of research in which it is hypothesized that genetics may affect bone metabolism. Better insight of genetic factors that are contributing to metabolic bone diseases would allow for focused nursing care and preventive interventions.

## 1. Introduction

Nursing research on the biological and psychosocial aspects of genetic health has developed in response to progress in genetic knowledge and nurses are expected by society to generate knowledge and provide health care that protects them from potential threats based on their genetic information [1,2]. Research in nursing is intended to provide options for the primary prevention of disease and health promotion, regardless of pathology or disease. Nurses have the skills to develop and lead research that addresses the relationship between genetic factors and health [3,4]. Increasing genetic knowledge and research capacity through interdisciplinary cooperation as well as the development of research resources will accelerate the rate at which nurses contribute to the knowledge about genetics and health [5]. Identifying associations between genotype and health conditions will enable nursing research to respond to health problems through innovative approaches. Current nursing genetics research opportunities include studying the ethical and social implications of genetic mechanisms of diagnosis, treatment and prevention of disease, understanding human responses to genetic aspects of health and disease, evaluating the application of genetic discoveries in health systems or even applying clinical knowledge in basic research [5]. Therefore, there are currently different fields in which knowledge can be expanded by research developed from the nursing field. Due to the nature of genetic research, it can be focused up to the level of the genes and the presence or not of some specific mutations in these genes. Nurses need to be more involved in all aspects of genetic testing such as sequencing and presentation of results to provide patients with the best available information and strategies to promote their health [6]. Here, we present an emerging field of research in which it is hypothesized that genetics related to breast cancer may affect bone metabolism. Better insight of genetic factors that contribute to metabolic bone diseases would allow for focused nursing care and preventive interventions.

## 2. Overview

Initially named as KAA0575 [7] and further renamed as Growth Regulation by Estrogen in Breast Cancer 1 (GREB1) [8], the GREB1 gene is a key intermediary for the estrogen-stimulated proliferation of breast cancer cells and the androgen-stimulated proliferation of prostate cancer cells. The GREB1 gene is remarkably conserved across species and is expressed in several tissues such as the brain, mammary gland, ovary, prostate, and endometrium [7,8,9,10,11]. With a specific role that persists confusion, the expanding evidence suggests that it plays a crucial function in the pathway of hormone responses in cancer cells, as GREB1 suppression diminishes cancer cell growth in in vitro models of cancer [10,12,13]. It seems that GREB1 is upregulated by the Estrogen Receptor 1 (ESR1) directly and not by one of its transcriptional targets as transcriptional induction of GREB1 is not dependent on protein synthesis [8,13,14].

GREB1 is located centromeric of marker D2S168 at 2p25.1 [15]. It has at least three isoforms, each transcribed from unique promoters leading to diverging 5′ untranslated regions [13]. Those isoforms of the human GREB1 protein are named respectively as GREB1a, GREB1b and GREB1c, encoding proteins of 1949 amino acids and through alternative splicing truncated proteins of 457 and 409 amino acids, respectively [8,13,16], little being known about putative differences in functions between the isoforms [17]. There is some evidence that may indicate that isoforms have differential effects on the expression of genes modulated by the estrogen receptor [17,18].

Although in vitro studies in breast cancer models showed that GREB1 siRNA-mediated suppression reduced cancer cell proliferation in the presence of 17-β estradiol (a major intermediary in the development and evolution of breast cancer [19]) and that GREB1 was involved in the estrogen stimulated cell growth through the estrogen/MYC/miR-26 axis [20], to date, the evidence indicates that GREB 1 expression correlates with good prognostic outcome in breast cancer patients [13,21]. It has been proposed that this association of GREB1 may be related to its decreased expression in endocrine-resistant cancers [13]. In young women with breast cancer, GREB1 expression was significantly higher than that observed in a cohort of older patients [22]. GREB1, in combination with other genes, also predicted recurrence of tamoxifen-treated breast cancer [23], as results confirmed in vitro as tamoxifen-resistant breast cancer cells showed a decrease in GREB1 expression while reestablishing GREB1 expression recovered sensitivity to tamoxifen [24].

There have been described other putative functions of GREB1 in various conditions such as ovarian cancer, prostate cancer, endometriosis, lung cancer, and hepatoblastoma. GREB1 is present in the majority of ovarian cancers of all primary histological subtypes [24]. In ovarian cancer, GREB1 was upregulated in mouse tumors treated with 17-β estradiol and was overexpressed in human ovarian cancers corresponding to human ovarian surface epithelium, implying a function for GREB1 in human ovarian tumor advancement [9]. In this sense, the inhibition of GREB1 expression in mouse ovarian cancer ascites (MAS) cell lines lowered their proliferation rate in vitro and raised survival time in mice engrafted with the MAS. 17-β estradiol promotes ovarian tumor growth in mouse models with GREB1 a key mediator of this process, so the mechanism involved probably implies the upregulation of collagen I expression [24]. Recently, it has also been proposed that GREB1 expression is highest in murine dysplastic ovarian surface epithelial and fallopian tube epithelium, suggesting that GREB1 may play a role in the initiation of tumor formation in both cell types [25]. Overall, the reported results indicate a major function for GREB1 in estrogen-dependent conditions in epithelial ovarian cancers [17].

GREB1 is expressed in proliferating prostatic tissue and prostate cancer, which is regulated by androgens, and suppression of GREB1 blocks androgen-induced growth, suggesting GREB1 may be firmly involved in prostate cancer progression [10,13]. In prostate cancer patients, tissue expression of GREB1 was associated with organ-confined prostate cancer and has been related with a positive prognosis [26]. However, recently it has been reported that GREB1 levels are elevated in the tumors of castration-resistant prostate cancer patients who have progressed undergoing enzalutamide treatment, providing evidence through xenograft models that GREB1 is required for in vivo enzalutamide resistance [27].

GREB1 may also have a function in other hormone-dependent cancers such uterine cancer [17]. An association between GREB1 upregulation and endometrial cancer [28] and uterine sarcomas [29,30] has been reported. Although there are no specific data about a role of GREB1 in testicular cancer, it has been associated with testicular functions [31,32] in murine models, which deserves further study from the cancer research point of view [17].

GREB1 also plays a role in endometriosis, which is 17-β estradiol-responsive [13]. In premenopausal women with endometriosis, increased expression of GREB1 has been reported [33]. A genome-wide association meta-analysis of 4604 endometriosis cases and 9393 controls of Japanese and European ancestry established an association of rs13394619 polymorphism in GREB1 with endometriosis [34]. Similarly, meta-analyses conducted on four genome-wide association studies showed that rs13394619 in GREB1 was associated with endometriosis with little evidence of population-based heterogeneity [35]. Expression of other polymorphisms rather than rs13394619 have also been associated with endometriosis risk [36,37,38] and infertility risk in women with endometriosis [39]. Although rs11674184 of GREB1 constitutes one of the most consistently associated SNPs with endometriosis in European ancestry populations, an absence of association with endometriosis in a cohort of 166 women has recently been reported [40].

There have been described associations of GREB1 with conditions rather than those corresponding to hormone-sensitive tissues. GREB1 and other genes have been proposed as putative salivary biomarkers for the non-invasive detection of lung cancer [41,42] as well as a target molecule of the Wnt/β-catenin pathway required for hepatoblastoma progression [43].

## 3. GREB1 Polymorphisms and Bone Mineral Density

The correlation between genetics and BMD (bone mineral density) is a growing subject of research where promising investigations can be pioneered from the nursing perspective. Different studies have focused on this relationship, primarily on genes that are directly related to bone metabolism [44,45,46,47]. The association between a gene related to breast cancer and its potential association with metabolic bone diseases raises a hypothesis that, if contrasted, can lead to an enormous potential for disease prevention in which, again, nursing may play key roles.

Beyond the cancer research, one of the growing fields of research involving GREB1 is its role in the BMD and derived its role in osteoporosis, which is a critical manifestation of low BMD. Estrogens and androgens control bone remodeling, so due to the close association of GREB1 expression with these hormones, a putative role of GREB1 in the maintenance of bone mineral density has been proposed. There has been estimated a potential contribution of genetic background to osteoporosis from between the 40–92% [48,49]. To date, several genes have been linked with BMD including parathyroid hormone receptor type 1, interleukin-6 receptor, interleukin 1 alpha, type II collagen A1, vitamin D receptor, and osteoprotegerin [50,51,52,53,54,55,56,57]; however, such results have been conflictive [45,46,47] and are probably population dependent. In Irish families, the study of 24 microsatellite markers by linkage studies reported the marker D2S168 from chromosome 2p22–25 to be related to both lumbar spine and femoral neck BMD, suggesting that genes in such regions were probably contributing to a predisposition to low BMD in such a population [57]. The fact is that GREB1 is located at such regions, and hence further research is being developed to assess its putative relationship with bone density [15].

To date, three studies have been performed covering three different populations and evaluating the role of GREB1 polymorphism in the BMD. The first association of common GREB1 polymorphism was communicated in the literature in 2009 from Hegarty and colleagues [58]. Since that communication, a total of two full studies involving Irish [15] and Beijing senile [59] populations and the communication of preliminary results in healthy African American children and two cohorts of Caucasian young adults [60] (the full original manuscript is in preparation) have been reported.

Two polymorphisms, rs10929757 and rs5020877, have been consistently studied in these reports. Hegarty and colleagues [15] reported data from two populations, one from a family based dataset that was used to find associations of polymorphism with BMD at the lumbar spine and/or the femoral neck and additionally, a sample of unrelated postmenopausal women to further study the hypothesized association. Their results confirmed evidence of association between GREB 1 polymorphism and BMD variation at the lumbar spine and the femoral neck. In the family based sample, a significant within-family association was observed for rs5020877 and BMD variation at both anatomical sites. The polymorphism rs5020877, however, was not associated to BMD in the postmenopausal sample [15]. In such a sample, the polymorphism rs10929757 (located downstream from rs5020877) was associated to femoral neck BMD variation (for each copy of the wild-type rs10929757 A allele, mean femoral neck BMD was increased) [15]. The lack of association of polymorphism rs5020877 in the family-based study may be due to a false positive due to genotyping error, population stratification, or inadequate control for multiple testing [61], according to Hegarty and colleagues [15].

Although the body of knowledge associating GREB1 polymorphism and BMD is growing, there is still a limited number of studies and populations involved. At this point, to hypothesize the function of GREB1 in this context is still speculative [15].

Both rs10929757 and rs5020877 polymorphism has recently being studied by Zheng and colleagues in a Chinese elderly population [59]. The study from Zheng and colleagues also deserved finding the associations with osteoporosis, so a group with osteoporotic fracture was studied. The polymorphisms rs10929757 and rs5020877 of the GREB1 gene were not associated to the BMD of the hip and lumbar spine, neither in the whole sample nor the female or male groups [59]. However, the polymorphism rs10929757 was associated with vertebral osteoporotic fracture in the male subgroup. Zheng and colleagues recognized in their study that the small sample size (*n* = 226) involved could lead to a Type II error (negative result).

Preliminary results about the association of GREB1 polymorphism and bone density in African American children have been reported by Lee and colleagues in 2019 [60]. Their final manuscript is under preparation, but preliminary results about the role of polymorphisms rs5020877 and rs10929757 on BMD measures in a cohort of African American children as well as two cohorts of Caucasian young adults indicated that there was a significant association between the rs5020877 and DXA z-scores of the left hip in the cohort of African American children. They did not find associations with BMD of the lumbar spine in African American children, but found a significant association with lumbar spine bone mineral content (BMC). Their study also found associations of rs10929757 and total body BMD in the cohort of African American children, with rs10929757 also significantly correlated with the DXA z-scores of the whole body, lumbar spine, and left hip in African American children in contrast to that previously reported in the study by Hegarty and colleagues [15]. Overall, the study of Lee and colleagues [60] further supports the hypothesized association between rs10929757 and bone density.

## 4. Concluding Remarks

The possibility of a genetic association between common GREB1 polymorphism rs10929757 and rs5020877 and bone mineral density has been recently suggested by several studies covering this putative association in different populations. Overall, the research on the role of GREB1 polymorphism rs10929757 and rs5020877 on BMD is very preliminary. At this point, the observations are not unanimous in observing such correlations, with variations in the anatomical sites and between populations. Negative associations regarding both polymorphism have been reported in Asian populations. However, there was no elevated difference in the frequency of the polymorphisms between the studied populations to date, so it does not seem that it is the responsibility of the discrepancies observed. It seems that the major incongruencies in the results reported between studies and populations would rely on the sample size and hence, further research is encouraged that should cover new populations and larger sample sizes [61]. Much has to be learned regarding the role of GREB1 polymorphisms to unveil individuals at risk of developing low bone mineral density and hence to individualize specific therapeutic approaches. Future research should confirm whether a particular SNP profile of the GREB1 gene may be a candidate for increased risk of low bone mass and thus benefit from preventive nursing interventions designed for potentially at-risk individuals. Nursing research with sufficient statistical power and diverse cohorts can contribute to replicate and contrast the early results reported thus far.
